# Host Lipids in Positive-Strand RNA Virus Genome Replication

**DOI:** 10.3389/fmicb.2019.00286

**Published:** 2019-02-26

**Authors:** Zhenlu Zhang, Guijuan He, Natalie A. Filipowicz, Glenn Randall, George A. Belov, Benjamin G. Kopek, Xiaofeng Wang

**Affiliations:** ^1^National Key Laboratory of Crop Biology, National Research Center for Apple Engineering and Technology, College of Horticulture Science and Engineering, Shandong Agricultural University, Tai'an, China; ^2^School of Plant and Environmental Sciences, Virginia Tech, Blacksburg, VA, United States; ^3^Fujian Province Key Laboratory of Plant Virology, Institute of Plant Virology, Fujian Agriculture and Forestry University, Fuzhou, China; ^4^Department of Biology, Hope College, Holland, MI, United States; ^5^Department of Microbiology, The University of Chicago, Chicago, IL, United States; ^6^Virginia-Maryland Regional College of Veterinary Medicine, University of Maryland, College Park, MD, United States

**Keywords:** lipid metabolism, phospholipids, membrane association, positive-strand RNA virus, viral RNA replication

## Abstract

Membrane association is a hallmark of the genome replication of positive-strand RNA viruses [(+)RNA viruses]. All well-studied (+)RNA viruses remodel host membranes and lipid metabolism through orchestrated virus-host interactions to create a suitable microenvironment to survive and thrive in host cells. Recent research has shown that host lipids, as major components of cellular membranes, play key roles in the replication of multiple (+)RNA viruses. This review focuses on how (+)RNA viruses manipulate host lipid synthesis and metabolism to facilitate their genomic RNA replication, and how interference with the cellular lipid metabolism affects viral replication.

## Introduction

**Figure 1:**
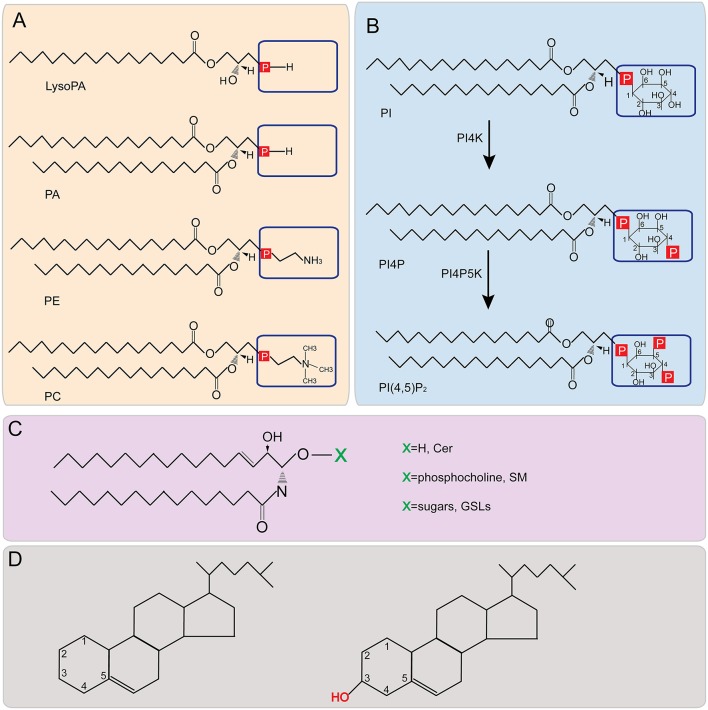
Structures of major lipids. **(A,B)** Phospholipid structure representations. Boxed areas indicate the different headgroups among various phospholipids. **(C)** Structure of sphingolipids. X represents different headgroups of different sphingolipids. **(D)** Basic structure of sterol (Left) and the structure of cholesterol (Right). PA, phosphatidic acid; PE, phosphatidylethanolamine; PC, phosphatidylcholine; PI, phosphatidylinositol; PI4P, phosphatidylinositol-4-phosphate; PI(4,5)P_2_, phosphatidylinositol-4,5-bisphosphate; PI4K, Phosphatidylinositol-4 kinase; PI4P5K, phosphatidylinositol 4-phosphate 5-kinase; Cer, ceramide; SM, sphingomyelin; GSL, glycosphingolipid.

Lipids are a diverse group of amphipathic or non-polar molecules essential for all cellular life forms. In eukaryotic cells, ~5% of genes are dedicated to the biosynthesis of thousands of lipid species (Sud et al., 2007; van Meer et al., 2008). Lipids are characterized by remarkable diversity in structure due to multiple factors, such as oxidation, reduction, and substitution, acyl chain composition, as well as modification by other groups, such as sugar residues (Fahy et al., 2011). In the classification system proposed by the lipid metabolites and pathways strategy (LIPID MAPS), lipids are classified into eight categories based on ketoacyl and isoprene groups: glycerophospholipids (also called phospholipids), sphingolipids, sterol lipids, fatty acids (FAs), glycerolipids, saccharolipids, polyketides, and prenol lipids (Fahy et al., 2011). Although each of the lipid types function differently, the complex lipid repertoire has three general cellular functions. First, some lipids, such as phospholipids, sphingolipids, and sterol lipids, serve as essential building components of cellular membranes (Figure 1). Second, some lipids are stored in lipid droplets (LDs) to serve as energy sources, e.g., triacylglycerol (TAG) and steryl ester (StE) that are produced from free FAs and sterols, respectively (Zweytick et al., 2000; Klug and Daum, 2014). Finally, some specific lipids, such as phosphatidic acid (PA) (Wang et al., 2006; Arisz et al., 2009), FAs (Glass and Olefsky, 2012; Huang et al., 2012), sterols (Wollam and Antebi, 2011), as well as glycerolipids (Drissner et al., 2007), and sphingolipids (SLs) (Wang et al., 2008; Gan et al., 2009), function as signaling molecules in multiple cellular processes.

As the major components of membranes, lipids play decisive roles in membrane flexibility and rigidity, which is critical for multiple morphological transformation-based membrane functions, including differentiation, division, and adaption to environment (Lipowsky, 2014; Nicolson, 2014). Several factors are involved in regulating membrane fluidity, including the degree of PL saturation, the length of acyl chains, and the number of sterols. In particular, the saturation degree of FAs plays a critical role in regulating membrane fluidity in both eukaryotic cells and bacteria (Cybulski et al., 2004; Mansilla et al., 2004; Ernst et al., 2016). The ratio of saturated to unsaturated acyl chains in phospholipids and SLs influences the packing of lipids and thus, the viscosity and water permeability of membranes (Lande et al., 1995). Cellular pathways involved in the saturation of FAs influence membrane fluidity. For example, the yeast OLE pathway, in which the *OLE1*-encoded Δ9-fatty acid desaturase (Ole1p) catalyzes the conversion of saturated FAs (SFAs) to unsaturated FAs (UFAs), is perhaps the best surveillance system of eukaryotic lipid saturation and membrane fluidity (Covino et al., 2016; Ernst et al., 2016; Ballweg and Ernst, 2017). Sterols are major factors in membrane fluidity regulation (Yeagle, 1985) and changes in the ratio of cholesterol to phospholipids alters membrane fluidity. Cholesterol increases membrane fluidity by interfering with the packing of acyl chains, resulting in inhibition of the transition to the solid gel state. Conversely, cholesterol can also rigidify membranes by reducing the flexibility of neighboring unsaturated acyl chains (Yeagle, 1985; Holthuis and Menon, 2014). Thus, the fluidity of membranes is dependent on the presence of specific lipids and sterols and their structure (i.e., saturation, length and number of acyl chains, and precise ratios). The remarkable flexibility of bilayer membranes makes possible the formation of multiple differently-shaped membranous compartments. Considering the key roles that lipids can play in changing membrane morphology, some pathogens, such as viruses, may remodel lipid metabolism and membrane structure to form a suitable microenvironment or membranous compartments for successful infection and replication.

**Figure 2:**
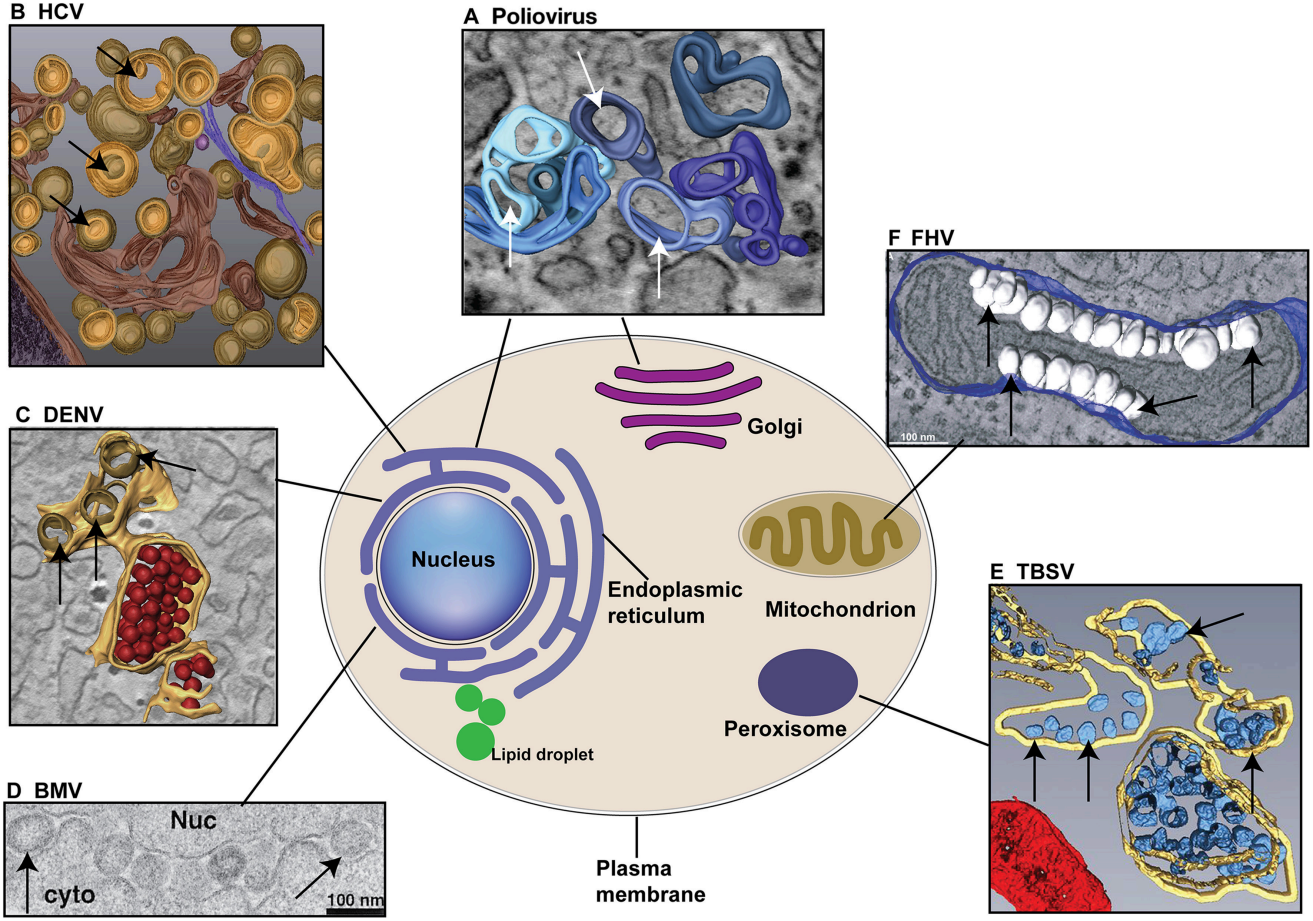
Different (+)RNA viruses explore specific cellular organelle membranes for VRCs assembly and genome replication. **(A)** Both ER and Golgi membranes are used for the formation of Poliovirus VRCs. The 3D model indicates the single-membrane tubules formed at early stage of poliovirus infection. **(B)** HCV utilizes ER membranes to form double-membrane vesicles (DMVs) as VRCs. **(C)** DENV replicates in association with ER membranes. ER membranes are in yellow, VRCs are in light brown. Note virions (in red) are produced near VRCs. **(D)** BMV invaginates the outer perinuclear ER membrane to assemble VRCs. Nuc: nucleus; cyto: cytoplasm. **(E)** TBSV replicates in association with the peroxisomal membranes and VRCs (blue) are formed inside multivesicular bodies (MVB, yellow membranes). **(F)** FHV invaginates the outer mitochondiral membranes (blue) to build VRCs (White). Arrows point to VRCs. **(A–F)** are reproduced with permission from Belov et al. (2012) in **(A)**; Romero-Brey et al. (2012) in **(B)**; Welsch et al. (2009) in **(C)**; Schwartz et al. (2002) in **(D)**; Fernandez de Castro et al. (2017) in **(E)**; and Kopek et al. (2007) in **(F)**.

**Figure 3:**
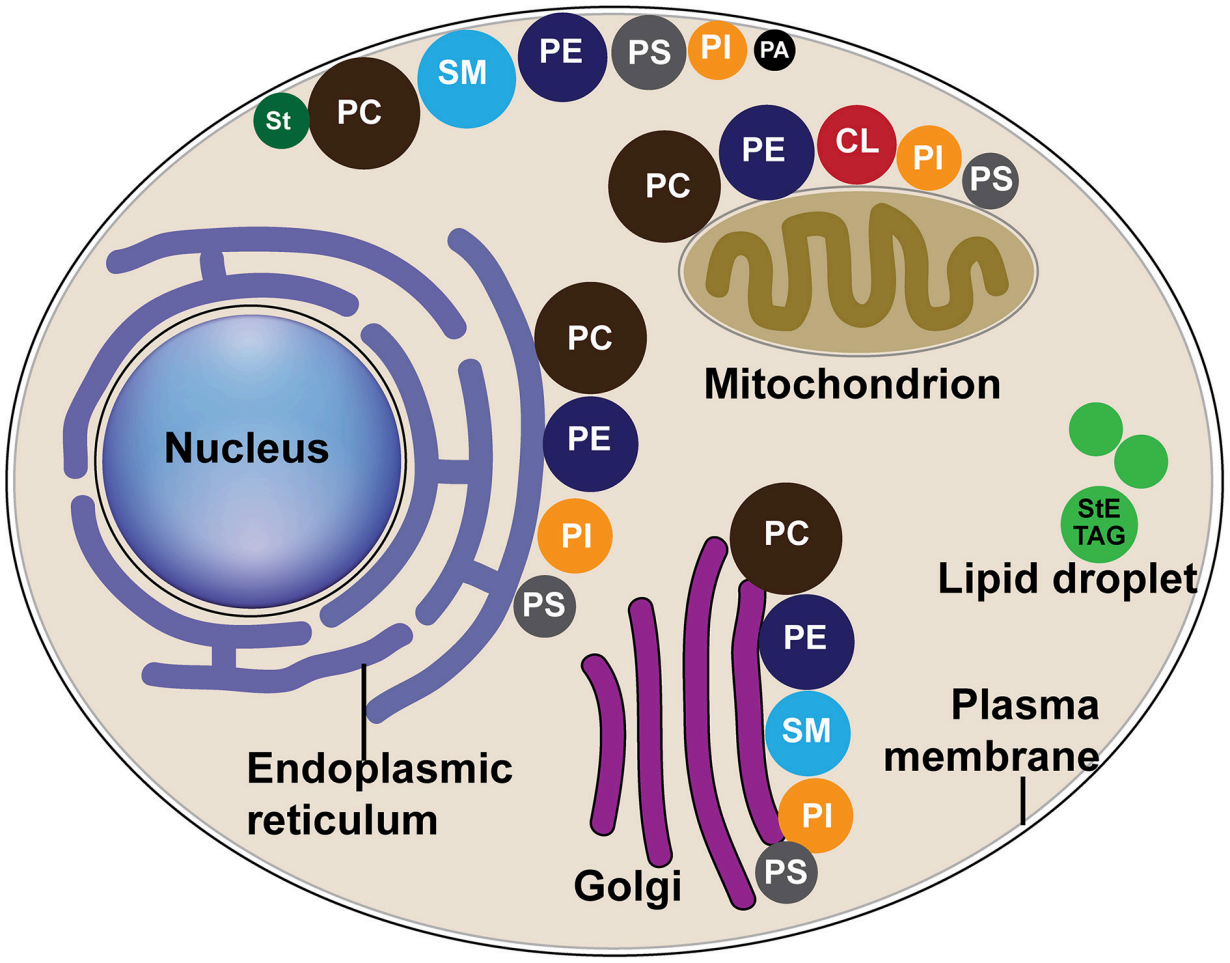
The distribution of major lipids in host organelle membranes. Different colors of circles represent different lipid classes, and the size of circles represent the percentage of one class of lipid to the total phospholipids, as modified from van Meer et al. (2008). CL, cardiolipin; PA, phosphatidic acid; PC, phosphatidylcholine; PE, phosphatidylethanolamine; PS, phosphatidylserine; PI, phosphatidylinositol; SM, sphingomyelin; St, sterol; StE, steryl ester; TAG, triacylglycerol.

Positive-stranded RNA viruses [(+)RNA viruses] are the most numerous of seven viral genetic classes and cause diseases in humans, animals, and plants. Despite genomic and structural diversity among various (+)RNA viruses, they share common features in genome replication (Ahlquist, 2006). These common features include the synthesis of a negative-strand RNA, asymmetric RNA synthesis of positive-strand over negative-strand RNA, and dependence on multiple host factors for genome replication, among others (Ahlquist, 2006; den Boon et al., 2010; Nagy and Pogany, 2012; Wang, 2015; Nagy, 2016). One key feature conserved among (+)RNA viruses of eukaryotes is that RNA genome synthesis occurs in tight association with remodeled organelle membranes (Figure 2 and Table 1), such as mitochondria (Rubino and Russo, 1998; Miller et al., 2001), chloroplast (Prod'homme et al., 2001), endosome (Grimley et al., 1968; Froshauer et al., 1988), peroxisome (Rubino and Russo, 1998; McCartney et al., 2005; Panavas et al., 2005; Pathak et al., 2008), or endoplasmic reticulum (ER) (Restrepo-Hartwig and Ahlquist, 1996, 1999; Suhy et al., 2000; Gosert et al., 2003). Although it is not well understood why viruses replicate in association with specific organellar membranes, differences in membrane lipid (Figure 3) (van Meer et al., 2008) and protein composition should play a critical role. For detailed information on distributions of different lipids in organelle membranes, readers are referred to van Meer et al. (2008).

**Table 1:** Specific lipid species required by different (+)RNA viruses for successful genome replication.

**Viruses**			**Required lipids**	**Viral replication sites**	**Lipids enriched**	**References**
**Family**	**Genus**	**Species**				
*Bromoviridae*	*Bromovirus*	Brome mosaic virus (BMV)	PC, PA, UFA	Outer perinuclear ER membrane	PC & PE	Lee et al., 2001; Lee and Ahlquist, 2003; Zhang et al., 2012, 2016; Zhang Z. et al., 2018
*Flaviviridae*	*Flavivirus*	Dengue virus (DENV)	PC, Sterol, FA	ER membrane	FA	Rothwell et al., 2009; Heaton and Randall, 2010; Heaton et al., 2010; Perera et al., 2012; Zhang J. et al., 2018
		West Nile virus (WNV)	SL, Sterol, FA	ER membrane	SL	Mackenzie et al., 2007; Heaton et al., 2010; Schuchman, 2010; Martin-Acebes et al., 2011, 2014, 2016; Aktepe et al., 2015
	*Hepacivirus*	Hepatitis C virus (HCV)	PC, PI4P, PI(4,5)P_2_, SL, sterol, FA,	ER membrane	PC, PI4P, sterol	Kapadia and Chisari, 2005; Sakamoto et al., 2005; Berger et al., 2009, 2011; Borawski et al., 2009; Arita et al., 2011; Reiss et al., 2011; Takano et al., 2011; Hirata et al., 2012; Nasheri et al., 2013; Khan et al., 2014; Lyn et al., 2014; Nguyen et al., 2014; Wang et al., 2014; Cho et al., 2015; Zhang et al., 2016
*Nodaviridae*	*Alphanodavirus*	Flock House virus (FHV)	PC	Outer mitochondrial membrane	N/A	Castorena et al., 2010
		Nodamura virus (NoV)	PE	Mitochondrial membrane	PE	Xu and Nagy, 2015
*Picornaviridae*	*Cardiovirus*	Mengovirus	PC	ER membrane	N/A	Plagemann et al., 1970; Schimrnel and Traub, 1987
		Encephalomyocarditis virus (EMCV)	Sterol, PI4P	ER membrane	PI4P, sterol	Dorobantu et al., 2015
*Picornaviridae*	*Enterovirus*	Poliovirus	PC, PI4P, PI(4,5)P_2_, Sterol	ER and Golgi membranes	PC, PI4P, PI(4,5)P_2_, sterol	Vance et al., 1980; Ilnytska et al., 2013; Nchoutmboube et al., 2013; Arita, 2014; Banerjee et al., 2018
		Coxsackievirus group B type 3 (CVB3)	Sterol, PI4P	ER and Golgi membranes	PI4P, sterol	Hsu et al., 2010; Ilnytska et al., 2013
		Human rhinovirus (HRV)	Sterol, PI4P	ER and Golgi membranes	Sterol, PI4P	Ilnytska et al., 2013; Roulin et al., 2014
	*Kobuvirus*	Aichi virus (AiV)	PI4P, sterol	ER membrane	PI4P, sterol	Sasaki et al., 2012; Ishikawa-Sasaki et al., 2014, 2018
*Tombusviridae*	*Dianthovirus*	Red clover necrotic mosaic virus (RCNMV)	PA	ER membrane	N/A	Hyodo et al., 2015
	*Tombusvirus*	Tomato bushy stunt virus (TBSV)	PE, PA, Sterol	Peroxisomal membrane	PE, sterol	Sharma et al., 2010; Barajas et al., 2014; Chuang et al., 2014; Xu and Nagy, 2015, 2017; Nagy, 2016
		Carnation Italian ringspot virus (CIRV)	PE	Mitochondrial membrane	PE	Xu and Nagy, 2015

**Figure 4:**
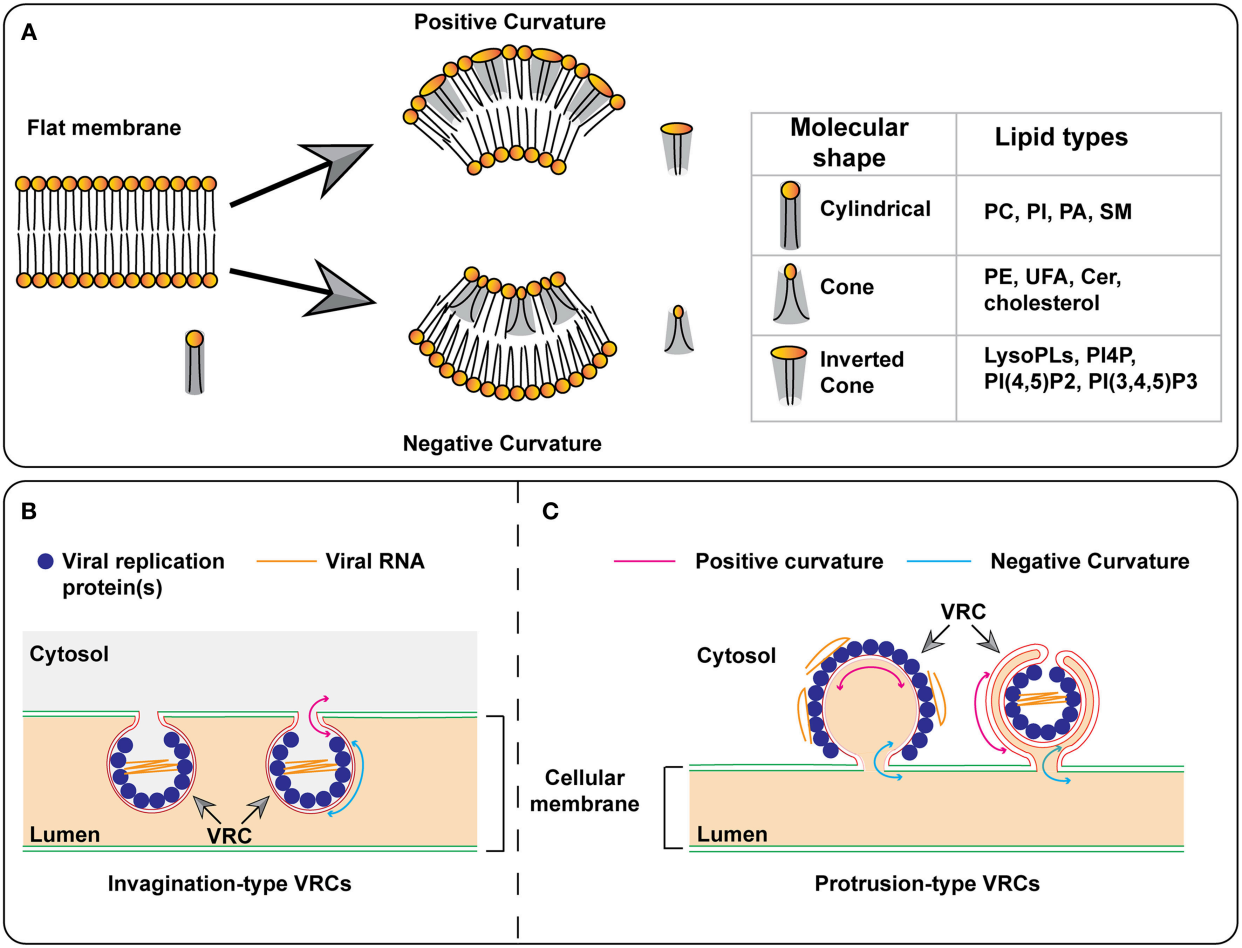
Models for the formation of membrane curvature and viral replication complexes. **(A)** The insertion of certain lipids with cone or inverted-cone shape generates negative or positive membrane curvature, respectively. **(B,C)** Models for the formation of invagination- and protrusion-type replication complexes. In the protrusion-type model, the VRC on the right represents the double-membrane vesicle (DMV). PC, phosphatidylcholine; PI, phosphatidylinositol; PI4P, phosphatidylinositol-4-phosphate; PI(4,5)P_2_, phosphatidylinositol-4,5-bisphosphate; PI(3,4,5)P_3_, phosphatidylinositol-3,4,5-trisphosphate; PA, phosphatidic acid; SM, sphingomyelin; PE, phosphatidylethanolamine; UFA, unsaturated fatty acid; Cer, ceramide; VRC, virus replication complex.

Regardless of the origin and host, (+)RNA viruses remodel cellular membranes to form membrane-bound viral replication complexes (VRCs) or mini-organelles (den Boon and Ahlquist, 2010; Belov and van Kuppeveld, 2012). VRCs can be morphologically categorized as invagination- or protrusion-type, based on whether the donor membrane bends away from or into the cytoplasm, respectively (Romero-Brey and Bartenschlager, 2014; Strating and van Kuppeveld, 2017) (Figure 4A). A negative membrane curvature means that the membrane invaginates away from the cytoplasm and thus, viral replication proteins reside and viral RNA synthesis occurs inside VRCs (Figure 4B). On the contrary, positive membrane curvature is created when membranes protrude into the cytoplasm and as such, viral RNA synthesis occurs on the surface of VRCs (Figure 4C, the model on the left). These protrusion VRCs can further fold as double-membrane vesicles (Figure 4C, the model on the right) so that viral replication occurs in a protected environment.

These membranous compartments aid (+)RNA virus replication by providing a physical scaffold for the assembly of replication machinery, including viral and host factors. Also, VRCs protect against host defense mechanisms including innate immunity sensors by shielding replication complexes containing double-strand RNA (dsRNA) replication intermediates. As major components of cellular membranes and signaling molecules, lipids have been demonstrated to be essential factors in many steps of the (+)RNA virus life cycle (Heaton and Randall, 2011; Belov and van Kuppeveld, 2012; Chukkapalli et al., 2012; Strating and van Kuppeveld, 2017). This review summarizes recent advances made in understanding the role of lipids in (+)RNA virus replication and how these viruses manipulate cellular lipid biosynthesis.

## Crucial Roles of Membrane Lipids in Genome Replication of (+)RNA Viruses

Phospholipids (PLs), SLs, and sterols function as structural constituents of membranes and are distributed throughout intracellular membranes (Figure 3) (Albert et al., 2002; van Meer et al., 2008). Interestingly, it was discovered that specific viruses not only require membranes on which to replicate, but also have a requirement or preference for a specific lipid composition of membranes. Recent discoveries have shown that to meet this requirement or preference, viruses manipulate host cell lipid metabolism, and trafficking pathways to ensure specific types of lipids are available.

The membranes associated with viral replication are rearranged into distinct structures, which can be in the form of small spherules with necks, double-membrane vesicles, membranous webs, and reticular layers. Although these morphologies are distinct, they all require the bending of membranes. Membrane bending and deformation are essential to many cellular processes (e.g., vesicle transport, locomotion) and cells have evolved multiple mechanisms to induce membrane curvature. Three ways that cells induce membrane deformation include: local enrichment of specific lipids, protein scaffolding, and binding protein insertion/interaction (McMahon and Gallop, 2005). As obligate intracellular parasites, viruses take advantage of these pathways for replication.

Each lipid molecule has a specific shape that can be categorized as cylindrical, conical, or inverted conical (Figure 4A) (Burger, 2000). While cylindrical lipids will produce a planar membrane, an enrichment of lipids with a conical or inverted-conical shape at one leaflet of the bilayer membrane will induce membrane deformation. This enrichment can be achieved by increased synthesis of a specific lipid and/or transport of lipids to a pre-existing membrane site. Phosphatidylcholine (PC), phosphotidylserine (PS), and sphingomyelin (SM) are cylindrical-shaped lipids. Phosphotidylethanolamine (PE) and phosphatidylinositol (PI) are conical-shaped lipids and can induce membranes with negative curvature. PI-4-phosphate (PI4P), PI(4,5)P_2_, and PI(3,4,5)P_3_ are inverted conical lipids that can lead to membranes with positive curvature. PC, PS, and SM can all form lipid bilayers, however, PE, PIs, and sterols cannot form lipid bilayers by themselves. In addition to the head groups, acyl chains can also play a role in membrane bending. The presence of a double-bond(s) in an acyl chain produces a “kink” that affects lipid packing, leading to changes in membrane shape and fluidity (Figure 4A). As described in this review, (+)RNA viruses can induce the synthesis of specific lipids as well as the transport of lipids to target sites. These viral mechanisms may be involved in the formation of the membrane rearrangements associated with VRCs.

Another characteristic of lipid molecules is the charge of the head group. PC and PE have a neutral charge while PS, PA, and PIs have negative charges. The charge of the lipid head group is often involved in the interaction and recruitment of proteins to specific sites on membranes. These interactions can range from simple electrostatic interactions to lipid-binding domains. Lipid headgroup charge is essential for multiple cellular membrane deformation processes and may play a role in VRC function and formation.

Therefore, local enrichment of specific lipids to the sites of viral replication can lead to changes in membrane shape, electrostatic charge, and the recruitment of membrane-binding proteins (Table 1). With these potential actions of lipids in mind, we will discuss each membrane lipid type individually and the evidence for its role in (+)RNA virus genome replication.

### Role of Phospholipids in the Genome Replication of (+)RNA Viruses

PLs are the most abundant and important structural lipids in eukaryotic cellular membranes (Figure 3). The major classes include PC, PE, PI, PS, and PA. The hydrophobic portion of PLs is a diacylglycerol (DAG), containing UFAs, or SFAs of varying lengths (Figures 1A,B). The hydrophilic moiety is a polar head group that determines the physical property and category of PLs (Daum et al., 1998) (Figures 1A,B).

### Phosphatidylcholine

**Figure 5:**
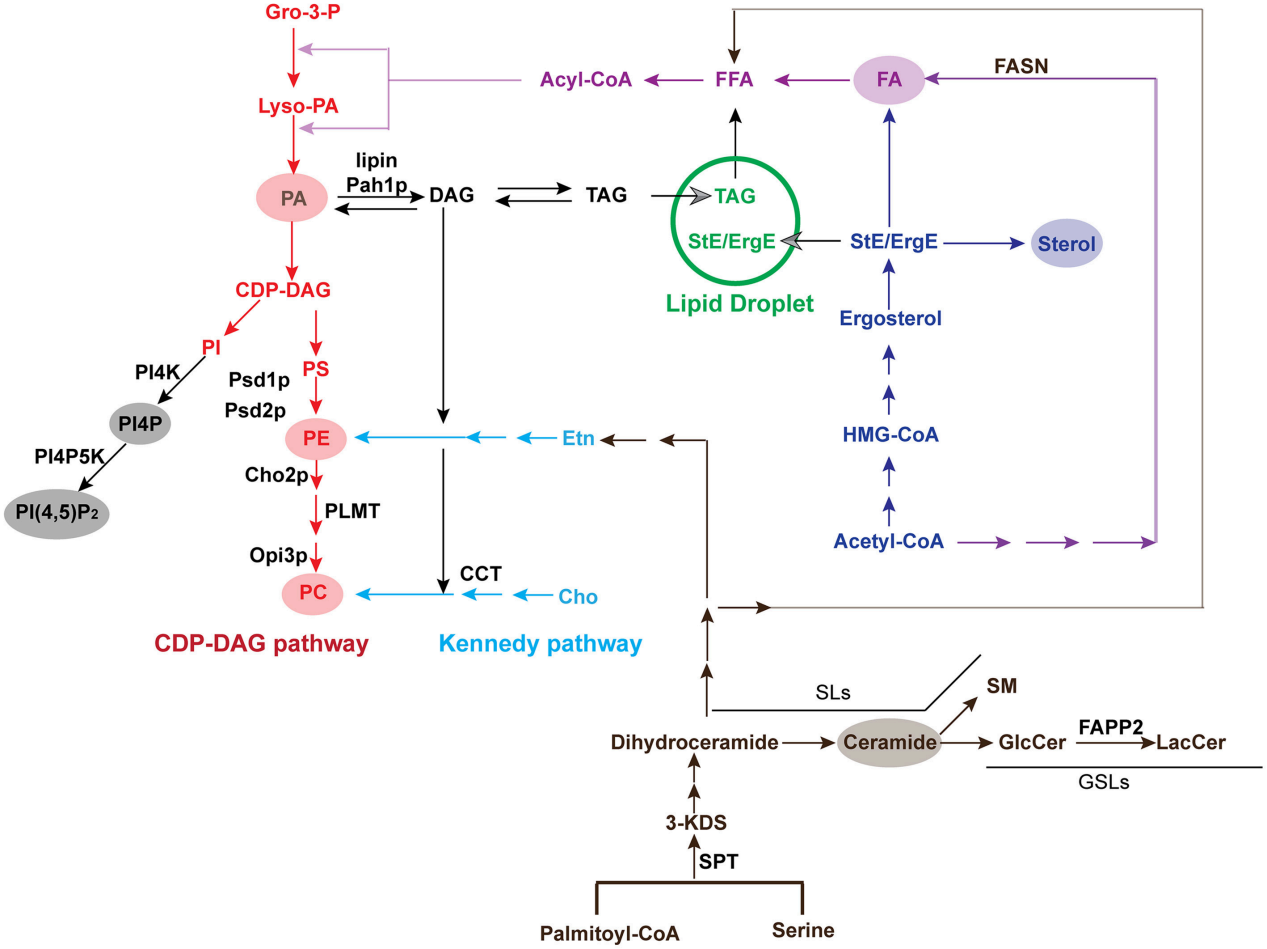
Pathways for the biosynthesis of major lipids. Key enzymes in pathways or those that are recruited by viruses are listed. The conversion from PE to PC is catalyzed by Cho2p and Opi3p in yeast but PLMT in mammals. Yeast or plant Pah1p or mammalian lipin converts DAG to PA. Gro-3-P, glycerol-3-phosphate; Lyso-PA, lysophosphatidic acid; PA, phosphatidic acid; CDP-DAG, cytidine diphosphate diacylglycerol; PI, phosphatidylinositol; PI4K, Phosphatidylinositol-4 kinase; PI4P, phosphatidylinositol-4-phosphate; PI4P5K, phosphatidylinositol 4-phosphate 5-kinase; PI(4,5)P_2_, phosphatidylinositol-4,5-bisphosphate; PS, phosphatidylserine; PE, phosphatidylethanolamine; PC, phosphatidylcholine; Etn, ethanolamine; Cho, choline; DAG, diacylglycerol; TAG, triacylglycerol; StE, steryl ester; ErgE, ergosterol ester; Acyl-CoA, acyl-coenzyme A; FFA, free fatty acid; FA, fatty acid; FASN, fatty acid synthase; HMG-CoA, 3-hydroxy-3-methylglutaryl-coenzyme A; SPT, serine palmitoyltransferase; 3-KDS, 3-ketodihydrosphingosine; GlcCer, glucosylceramide; LacCer, lactosylceramide; SM, sphingomyelin; SL, sphingolipid; GSL, glycosphingolipid; FAPP2, four-phosphate adaptor protein 2.

Phosphatidylcholines are the most abundant phospholipids in eukaryotic cellular membranes, making up more than 50% of total membrane PLs (Figure 3) (van Meer et al., 2008). In eukaryotes, PC is produced from either the Kennedy pathway or the CDP-DAG (cytidine diphosphate-diacylglyerol) pathway (Henry et al., 2012) (Figure 5). In most mammalian cell types, PC is produced from the Kennedy pathway (also termed salvage or CDP-choline) where exogenous choline is converted to PC through three steps catalyzed by choline kinase, CTP:phosphocholine cytidylytransferase (CCT) and CDP-choline:1,2-diacylglycerol cholinephosphostransferase (Cole et al., 2012). Conversion of PE to PC in mammals only occurs in liver cells (Li and Vance, 2008). In yeast, PC is mainly synthesized through the CDP-DAG pathway, in which PE is converted to PC by three sequential methylation steps (Gaynor and Carman, 1990): the first step is catalyzed by *CHO2*-encoded PE methyltransferase Cho2p (choline requiring 2) and the last two are catalyzed by *OPI3*-encoded phospholipid methyltransferase Opi3p (overproducer of inositol 3) (Kodaki and Yamashita, 1987, 1989; Summers et al., 1988); (Mcgraw and Henry, 1989).

It has been reported that PC synthesis is significantly upregulated by multiple (+)RNA viruses, such as brome mosaic virus (BMV) (Zhang et al., 2016), Flock House virus (FHV) (Castorena et al., 2010), Dengue virus (DENV) (Perera et al., 2012), poliovirus (Vance et al., 1980; Nchoutmboube et al., 2013), and mengovirus (Plagemann et al., 1970; Schimrnel and Traub, 1987). BMV-infection led to an increase of ~25% in the absolute amount of PC in both barley and yeast cells (Zhang et al., 2016). Poliovirus and mengovirus, both belonging to *Picornaviridae*, promoted choline incorporation to increase the rate of PC synthesis in virus-infected cells (Plagemann et al., 1970; Vance et al., 1980). Specifically, poliovirus stimulated the import of fatty acids into host cells by stimulating the activity of host long-chain acyl-CoA synthetase (Nchoutmboube et al., 2013), and thus, promoted the PC biosynthesis catalyzed by CCT (Vance et al., 1980). In addition, FHV and DENV stimulate PC synthesis by 35% and ~2-fold in virus-infected *Drosophila* and mosquito cells, respectively (Castorena et al., 2010; Perera et al., 2012). For FHV, downregulation of CCT in *Drosophila* cells suppresses FHV RNA replication by 40–65% (Castorena et al., 2010).

A key question related to virus-promoted PC accumulation is where the newly synthesized PC is localized, whether globally distributed or associated with VRCs in infected cells. Using a monoclonal antibody that specifically recognizes PC but no other PLs (Nam et al., 1990; Fujimoto et al., 1996), PC was found to be enriched at the viral replication sites in BMV-replicating yeast and barley cells (Zhang et al., 2016). It is worth noting that the viral replication site-enriched PC accumulation is a common feature among a group of diverse (+)RNA viruses, including BMV, hepatitis C virus (HCV) and poliovirus, which belong to alphavirus-, flavivirus-, and picornavirus-like superfamily, respectively (Zhang et al., 2016; Banerjee et al., 2018).

The BMV replication protein 1a interacts with and recruits Cho2p, the PE methyltransferase, to the viral replication sites and promotes PC synthesis to facilitate viral replication in yeast cells (Zhang et al., 2016). Deleting *CHO2* resulted in the formation of VRCs that are 25% larger than those in wt cells and reduced BMV replication up to ~30-fold (Zhang et al., 2016). Conversely, overexpression of *CHO2* promoted viral replication by 70%, indicating a critical role of PC in BMV VRC formation and viral RNA replication (Zhang et al., 2016; Zhang Z. et al., 2018). These results suggest that BMV-promoted PC accumulation is primarily due to the synthesis at viral replication sites, rather than trafficking from cellular pools. For HCV, however, it remains to be elucidated whether accumulated PC is newly synthesized at viral replication sites, as is the case with BMV, or redistributed to the VRC. For poliovirus, expression of the 3CD protein (the precursor of 3C and 3D^pol^) alone is able to induce membrane rearrangements and PC synthesis, suggesting viral replication is not required to stimulate PC accumulation (Banerjee et al., 2018). It was also recently demonstrated that the activity of the poliovirus protease 2A is important for the relocalization of CCTα, the major isoform of the CCT enzymes in mammalian cells, from the nucleus to the viral replication sites. CCTα was shown to be essential for the enhanced PC synthesis in poliovirus-infected cells (Viktorova et al., 2018).

Labeling and identifying specific lipids within cells is more difficult than other targets such as proteins or nucleic acids. However, techniques have emerged that allow for the labeling and imaging of choline containing lipids, specifically PC, through the use of choline containing analogs. These choline analogs can be incorporated into PC in place of choline and contain functional groups for the tagging of PC molecules (Jao et al., 2009). This technique was used to show that PC colocalizes with the viral replication sites of poliovirus (Zhang et al., 2016) and could prove useful in understanding localization of PC in other (+)RNA virus-infected cells.

### Phosphatidylethanolamine

PE is another abundant class of PL and is synthesized from both the CDP-DAG and Kennedy pathways in eukaryotes (Henry et al., 2012) (Figure 5). Similar to PC, PE is predominantly produced via the CDP-DAG pathway in yeast cells, where PS is converted to PE by *PSD1*-encoded PS decarboxylase (Psd1p) at the inner mitochondrial membrane (Clancey et al., 1993; Trotter et al., 1993). A small portion of PE molecules in association with the Golgi or vacuoles are decarboxylated by the *PSD2*-encoded enzyme (Trotter et al., 1993, 1995; Voelker, 2003). Similar to PC, PE is primarily synthesized via the Kennedy pathway in higher eukaryotes.

PE was reported to play key roles in genomic replication of tomato bushy stunt virus (TBSV) and carnation Italian ringspot virus (CIRV), which both belong to the family *Tombusviridae* (Xu and Nagy, 2015). In TBSV-replicating yeast and plant cells, PE levels increased significantly. In addition, PE was redistributed to viral replication sites by TBSV replication protein p33 (Xu and Nagy, 2015), which interacted with and recruited host endosomal Rab5 small GTPase to facilitate the enrichment of PE to the viral replication sites via the actin network (Xu and Nagy, 2016). Deleting *CHO2* dramatically promoted TBSV replication due to increased PE levels (Xu and Nagy, 2015), but inhibited BMV replication by blocking PC synthesis (Zhang et al., 2016). This suggests that different lipid microenvironments support efficient replication of different (+)RNA viruses. In addition, PE was redistributed to viral replication sites in BMV-replicating yeast cells (Zhang et al., 2016). However, it is not clear whether the increased PE serves as a substrate for PC and/or is involved in the formation of BMV VRCs. Along with TBSV and CIRV, PE was also involved in replication of Nodamura virus (NoV), a virus from the *alphanodaviridae* family (Xu and Nagy, 2015). In contrast, PE does not appear to play a substantial role in the replication of the closely related *alphanodavirus* FHV in *Drosophila* cells (Castorena et al., 2010). This could be due to the different lipid synthesis pathways that predominate in specific cell types.

The NoV work regarding the role of PC was performed in yeast cells, where PC is primarily produced from PE via the CDP-DAG pathway, while the FHV work was performed in *Drosophila* cells, where the Kennedy pathway predominates and the methyltransferase enzyme(s) required to convert PE to PC has not been documented. This work brings up interesting questions regarding lipid-type specificity vs. overall membrane composition as discussed in Conclusions, Cautions, and Future Directions.

PE is a cone-shaped lipid with a relatively small, polar head group (Burger, 2000; van Meer et al., 2008). Due to its shape and polar head group, PE may facilitate the induction of negative curvature in the VRCs of viruses that rely on this PL for replication, such as BMV and TBSV. Both BMV and TBSV invaginate host intracellular membranes away from the cytoplasm to form spherules with a negative curvature at the main body (Figure 4B). For BMV, it invaginates the outer perinuclear ER membranes into the ER lumen while TBSV induces the invagination of the peroxisome membrane. The involvement of PE in BMV and TBSV replication suggests that PE contributes to this negative curvature in the VRCs of these two viruses.

### Phosphatidylinositol Derivatives

PI is synthesized by combining the phosphatidyl portion from the CDP-DAG pathway with inositol (Paulus and Kennedy, 1959; Fischl and Carman, 1983) (Figure 1). PI is the precursor of various phosphoinositides that have been demonstrated to play key roles in intracellular signaling pathways (Nishizuka, 1984; Cantley, 2002; Toker, 2002) and vesicular membrane trafficking (Itoh et al., 2001).

Phosphatidylinositol-4-phosphate (PI4P) is formed by esterifying the -OH group at the 4-position of the inositol ring with a phosphate group (Figure 1B). This esterification is catalyzed by Phosphatidylinositol-4 kinase (PI4K), a conserved enzyme from yeast to humans which has two types (II, III), each containing two isoforms (α, β) (Balla and Balla, 2006) (Figure 1B). PI4P plays a key role in the replication of multiple (+)RNA viruses, including HCV (Berger et al., 2009, 2011; Borawski et al., 2009; Hsu et al., 2010; Arita et al., 2011; Reiss et al., 2011) and enteroviruses (Hsu et al., 2010). HCV infection promotes the accumulation of PI4P, which partially colocalizes with ER membranes, while in uninfected cells PI4P is mainly located in Golgi bodies (Berger et al., 2011). Three different research groups found that the HCV nonstructural protein 5A (NS5A) interacted with and recruited PI4KIIIα to the viral replication compartments and activated PI4KIIIα enzymatic function to produce PI4P (Berger et al., 2009, 2011; Reiss et al., 2011; Tai and Salloum, 2011). PI4P is involved in generating and maintaining the integrity of membranous webs, which are believed to be HCV VRCs (Berger et al., 2009, 2011; Reiss et al., 2011). It is clear that the enzymatic activity of PI4KIIIα is required for HCV replication because cells expressing a catalytically inactive PI4KIIIα cannot support HCV replication (Berger et al., 2009). HCV infection increases the PI4P accumulation but not the abundance of PI4KIIIα and in addition, inactivation of PI4KIIIα enzymatic activity resulted in the abnormal morphology and reduced numbers of HCV VRCs (Berger et al., 2011; Reiss et al., 2011). However, recent results demonstrated that high levels of PI4KIIIα in hepatoma cells inhibited the replication of non-adapted HCV isolates, whereas adaptive mutations of NS5A and NS5B prevented PI4KIIIα overactivation to promote HCV replication (Harak et al., 2017). These results suggest the essential roles of PI4KIIIα in the replication and adaption of HCV in different cellular environments (Harak et al., 2017). Different from HCV, enteroviral replication protein 3A primarily recruits PI4KIIIβ for viral replication (Hsu et al., 2010). In fact, PI4KIIIβ was physically associated with the VRCs during viral infection. Enteroviral replication levels could be regulated by PI4P accumulation at the replication organelles, which may be due to the direct binding of the viral RNA polymerase 3D^pol^ and/or protease 3CD to PI4P (Hsu et al., 2010; Banerjee et al., 2018). For Aichi virus (AiV), another member of the family *Picornaviridae*, the recruitment of PI4KIIIβ is mediated by acyl-coenzyme A binding domain containing 3 (ACBD3). ACBD3 interacts with both PI4KIIIβ and AiV viral proteins to form a viral protein/ACBD3/ PI4KIIIβ complex to produce PI4P (Sasaki et al., 2012; Ishikawa-Sasaki et al., 2014). How PI4P is directly involved in HCV and poliovirus replication is not entirely clear. One possibility is that PI4P may be a component of HCV VRCs because PI4P can induce curvature of local membranes (McMahon and Gallop, 2005). Another possibility is that PI4P may act as a bridge to recruit other proviral host factors or even viral components to viral replication sites. PI4P has been reported to specifically bind to disrupted four-phosphate adaptor protein 2 (FAPP2) (Khan et al., 2014), oxysterol-binding protein (OSBP) (Amako et al., 2009; Wang et al., 2014), ceramide transfer protein (CERT) (Amako et al., 2011), and Golgi phosphoprotein 3 (GOLPH3) (Bishé et al., 2012), some of which are tightly involved in (+)RNA virus replication (Lemmon, 2008; Santiago-Tirado and Bretscher, 2011).

Phosphatidylinositol-4,5-bisphosphate (PI(4,5)P_2_) is another PI derivative that is modulated by poliovirus (Banerjee et al., 2018) and involved in HCV replication (Cho et al., 2015). PI(4,5)P_2_ accumulates at viral replication sites in HCV infected cells (Cho et al., 2015). The HCV nonstructural protein NS5A preferentially binds to PI(4,5)P_2_ through a novel motif named Basic Amino Acid PI(4,5)P_2_ Pincer (BAAPP) within its N-terminal amphipathic helix (Cho et al., 2015). Substitutions in BAAPP that blocked its binding to PI(4,5)P_2_ severely attenuated viral replication, suggesting the importance of the domain (Cho et al., 2015). It was further shown that binding to PI(4,5)P_2_ induced a conformational change of NS5A BAAPP domain and promoted the interaction between NS5A and the host proviral factor TBC1D20, a guanosine triphosphate activating protein for Rab1 (Sklan et al., 2007a,b; Cho et al., 2015). A putative BAAPP domain is present in multiple viral replication proteins such as 2C proteins of human rhinoviruses and enteroviruses, the HCV NS4B protein, as well as the core protein of DENV and Japanese encephalitis virus (Cho et al., 2015). However, it is yet to be tested whether these putative BAAPP domains bind to PI(4,5)P_2_. PI(4,5)P_2_ is the product of PI4P catalyzed by phosphatidylinositol 4-phosphate 5-kinase (PI4P5K) (Hay et al., 1995) (Figures 1B, 3). It is possible that PI(4,5)P_2_ is produced from the enriched PI4P pool at viral replication sites or redistributed from other cellular pools. Of note, full HCV replication, but not the expression of NS5A alone, promoted the co-localization of PI(4,5)P_2_ and NS5A (Cho et al., 2015). This is different from poliovirus 3CD, which not only promoted the levels of accumulated PI(4,5)P_2_ but also the co-localization of PI(4,5)P_2_ and 3CD (Banerjee et al., 2018). Thus, the role of PI(4,5)P_2_ in viral replication and how HCV and poliovirus modulate its synthesis may be different.

### Phosphatidic Acid

In eukaryotic cells, PA is a major precursor for all membrane PLs. PA can be produced via several pathways by multiple enzymes, such as phospholipase D (PLD), diacylglycerol kinase (DGK), and enzymes involved in the *de novo* synthesis from glycerol-3-phosphate (Wang et al., 2006) (Figure 5). In addition to serving as a precursor for PLs, PA is also an important signaling molecule involved in many cellular processes and responses to biotic and abiotic stresses in mammals, plants, and microorganisms (Wang et al., 2006).

In yeast, PA is either converted to PLs in the CDP-DAG pathway or converted to DAG by *PAH1*-encoded phosphatidate phosphatase (termed Pah1p in yeast and lipins in mammals). DAG is primarily converted to TAG and stored in LDs in the absence of free choline (Han et al., 2006; Henry et al., 2012) (Figure 5). Several pieces of evidence have shown that PA regulates (+)RNA viral replication (Chuang et al., 2014; Zhang Z. et al., 2018). For instance, deletion of the sole *LPIN* ortholog in yeast, *PAH1*, increases PA levels. The high-level PA, in turn, induces the extension of nuclear membranes and promotes the synthesis of PLs (Han et al., 2008). In *PAH1*-deletion cells, TBSV readily switches replication sites from peroxisomal membranes, where it replicates normally, to the extended nuclear membranes for a much-enhanced viral replication than that in wild-type (wt) cells (Chuang et al., 2014). Disrupted Pah1p also promotes BMV replication by ~2-3-fold. However, increased levels of PLs, rather than the extended nuclear membrane, is responsible for the enhanced BMV RNA replication, indicating that different viruses take advantage of different cellular responses to high PA levels (Zhang Z. et al., 2018). PA has also been shown to be directly involved in the replication of red clover necrotic mosaic virus (RCNMV), a member of the same *Tombusviridae* family of plant viruses as TBSV (Hyodo et al., 2015). RCNMV infection significantly increases the accumulation of PA levels via recruitment of PLDα and PLDβ (Hyodo et al., 2015). Host PLDα and PLDβ, which hydrolyze PE and PC to generate PA, are recruited to viral replication sites, probably through the binding with viral replicase p88^pol^ (Hyodo et al., 2015). Knocking down the expression of either gene encoding PLDs or disrupting their activity via an inhibitor such as *n*-butanol, greatly inhibits RCNMV replication (Hyodo et al., 2015). The RCNMV auxiliary replication protein p27 binds PA directly and addition of exogenous PA stimulates viral RNA synthesis in plant protoplasts (Hyodo et al., 2015). How specifically PA is involved in RCNMV RNA synthesis, whether it stimulates replicase activity via binding to p27 or recruits PA effector proteins to VRCs, needs further investigation. Nevertheless, PA is involved in viral replication possibly via multiple mechanisms for different viruses: (1) regulates the synthesis of PLs; (2) remodels ER and/or nuclear membranes to provide more room for VRC assembly; (3) stimulates activity of viral replication proteins; or (4) recruits PA effector proteins that might promote viral VRC assembly or functions.

Although Pah1p restricts BMV and TBSV replication, it needs to be noted that lipin1 protein is a proviral factor for HCV replication (Mingorance et al., 2018). *LPIN1* expression is promoted during HCV replication. Down-regulation of the gene expression of *LPIN1*, but not *LPIN2*, inhibited the formation of VRCs and thus, HCV replication. However, it is not clear whether PA, DAG, or other specific phospholipid(s) alteration affects HCV VRC formation in lipin1-deficient cells (Mingorance et al., 2018).

### Roles of Sphingolipids in (+)RNA Virus Replication

Sphingolipids (SLs) are another class of membrane structural lipids. They are enriched in plasma membrane (van Meer et al., 2008) and play critical roles in signaling transduction (Hanada et al., 1992; Pinto et al., 1992; Adachi-Yamada et al., 1999). SLs are *de novo* synthesized from serine and palmitoyl-CoA in ER membranes. The first and rate-limiting step of SL synthesis is catalyzed by serine palmitoyltransferase (SPT) to produce 3-ketodihydrosphingosine (Merrill, 1983) (Figure 5). Numerous SLs, such as sphingomyelin (SM), glucosylceramide (GlcCer), and galactosylceramide (GalCer), are generated in the Golgi apparatus by using the major precursor, ceramide (Figures 1C, 3) (Yamaji and Hanada, 2015).

The requirement of SLs in VRC assembly and genome replication of (+)RNA viruses is best demonstrated in HCV. It has been reported that HCV increases SL levels in infected host cells (Roe et al., 2011; Hirata et al., 2012; Khan et al., 2014). Further investigations have shown that viral nonstructural protein 5B (NS5B), the RNA-dependent RNA polymerase (RdRp) of HCV, contains a helix-turn-helix sphingolipid-binding motif (Glu230-Gly263) in its finger domain (Sakamoto et al., 2005; Hirata et al., 2012). Both the synthesized peptide containing sphingolipid-binding motif and the purified NS5B protein are directly bound to SM *in vitro* (Sakamoto et al., 2005). The binding of NS5B to SLs facilitates localization of NS5B to raft domains or detergent-resistant membrane (DRM), where HCV assembles its VRCs (Shi et al., 2003; Aizaki et al., 2004). Suppressed enzymatic activity of SPT reduces levels of SLs and results in an inhibited HCV replication in host cells (Sakamoto et al., 2005; Umehara et al., 2006) or in chimeric mice harboring human hepatocytes (Hirata et al., 2012). A SPT inhibitor disrupts the association of NS5B with DRM fractions and thus, inhibits HCV replication (Hirata et al., 2012). Conversely, NS5B's RdRp enzymatic activity and RNA synthesis are stimulated by certain SM species (*d*18:1–16:0 and *d*18:1–24:0), which are present in DRM with the highest level compared to other SM species (Hirata et al., 2012). Additionally, HCV replication is impeded by pharmacological inhibitors of GlcCer synthase or when FAPP2 is knocked down in Huh 7.5 cells (Khan et al., 2014). GlcCer synthase converts ceramides to GlcCer and FAPP2 plays a crucial role in the transport of GlcCer from *cis*- to *trans*-Golgi, where GlcCer is further converted to lactosylceramides (Figure 5). FAPP2 possesses a pleckstin homology (PH) domain and a glycolipid transfer protein (GLTP) domain (Godi et al., 2004; D'Angelo et al., 2007). The PH domain binds to PI4P and Arf1 GTPase and the GLTP domain binds to GlcCer (Godi et al., 2004; D'Angelo et al., 2007; Cao et al., 2009). Each domain is required for HCV genome replication (Khan et al., 2014). FAPP2 is redistributed to viral replication sites during HCV infection and its depletion results in the altered localization of replicase and the formation of abnormal VRCs (Khan et al., 2014).

SLs are also involved in West Nile virus (WNV) genome replication. WNV infection significantly increases levels of a number of SL species, such as SM, dihydroceramide, and ceramide (Martin-Acebes et al., 2014). Increased SM levels, either by inactivating acid sphingomyelinase (Schuchman, 2010), or supplementing ceramide in cell cultures, results in higher levels of WNV replication (Martin-Acebes et al., 2016). It has been further demonstrated that SM is enriched at viral replication sites in WNV-infected cells (Aktepe et al., 2015; Martin-Acebes et al., 2016). Surprisingly, although increased ceramide production through *de novo* synthesis is required for WNV replication, it is an inhibitory factor for DENV replication (Aktepe et al., 2015), suggesting that different viruses, even from the same genus, may have different uses for ceramide and other lipids for replication.

### Role of Sterol in (+)RNA Virus Replication

While both PLs and SLs are polar membrane lipids, sterols are the major non-polar membrane lipids that are required for the integrity of cellular membranes (Figure 1). Sterols are present predominantly as cholesterols in mammals (Figure 1D), stigmasterol, sitosterol and campesterol in plants, and ergosterols (Erg) in yeasts and fungi (Daum et al., 1998). Sterols are first synthesized at ER membranes and rapidly transported to their destination organelles. As such, sterol levels are lowest in ER membranes. Higher levels of sterols are present in organelles associated with secretory pathways, with the highest levels seen in the plasma membrane, which harbor ~90% of the free sterols of each cell (Lange et al., 1989; Zinser et al., 1993; van Meer et al., 2008). Sterols can be transported among different organelles in a vesicular or a non-vesicular manner. The non-vesicular transportation of lipids is through membrane contact sites (MCSs), where membranes from different organelles come into close apposition (Helle et al., 2013). This transportation is mediated by soluble lipid binding proteins, such as the well-investigated OSBP and the OSBP-related proteins (ORPs) (Maxfield and Menon, 2006; Mesmin et al., 2013).

Free cholesterol is enriched in viral replication complexes of several enteroviruses, including poliovirus, Coxsackievirus group B type 3 (CVB3), human rhinovirus (HRV), and echoviruses, along with an inhibited cholesterol esterification and depleted LDs in enterovirus-replicating cells (Ilnytska et al., 2013). The clathrin-mediated endocytosis is modulated by enteroviruses, most likely via viral protein 2BC, to enhance the intake of cholesterol into cells and the enrichment of intracellular free cholesterol during viral infection. Free cholesterol is further transported to VRCs via Rab11-containing recycling endosomes. The enrichment of free cholesterol to VRCs is achieved by an interaction between Rab11 and viral replication protein 3A. It has been further shown that the processing of 3CD is promoted by the enhanced cholesterol levels, but how the processing of 3CD could be regulated by cholesterol is not known (Ilnytska et al., 2013).

OSBPs, being located at the ER-Golgi MCSs, tether ER and Golgi membranes by interacting with vesicle-associated membrane protein-associated protein A (VAP-A) from ER membranes and PI4P from Golgi membranes. These OSBPs simultaneously shuttle cholesterols to the Golgi and PI4P to ER membranes (Mesmin et al., 2013). The OSBP/PI4P-dependent cholesterol enrichment pathway is known to be exploited by several diverse (+)RNA viruses, including poliovirus (Arita, 2014), HCV (Wang et al., 2014), HRV (Roulin et al., 2014), AiV (Ishikawa-Sasaki et al., 2018), and encephalomyocarditis virus (EMCV) (Dorobantu et al., 2015). Specifically, PI4KIIIβ is recruited by poliovirus protein 2BC (Arita, 2014) or HRV 2B or 2BC (Roulin et al., 2014, 2018) to promote the biosynthesis of PI4P and the recruitment of OSBP to virus-induced membranous structures or VRCs. These recruitments promote the accumulation of free cholesterol. On the other hand, HCV NS5A and EMCV 3A interact with PI4KIIIα to promote PI4P production and OSBP recruitment. The result of this interaction is also an increased transport of cholesterol to VRCs, leading to efficient viral genomic replication (Reiss et al., 2011; Wang et al., 2014; Dorobantu et al., 2015). In addition to this pathway, HCV also utilizes lipid transfer proteins, such as Niemann-Pick-type C1 (NPC1), to facilitate the recruitment of cholesterol to viral replication organelle via MCSs (Stoeck et al., 2018). Both of the two pathways may explain why cholesterol is highly enriched in HCV replication compartments (Paul et al., 2013). Although the OSBP/PI4P pathway is required for HCV replication, DENV replicates independently of either PI4KIIIα or OSBP (Martin-Acebes et al., 2011; Wang et al., 2014). In addition, disruption of PI4K and/or OSBP, either by RNA interference or pharmacological inhibitors, inhibits genome replication of multiple viruses including HCV, poliovirus, EMCV, and enteroviruses (Reiss et al., 2011; Arita, 2014; Wang et al., 2014; Dorobantu et al., 2015; Strating et al., 2015).

In addition to the OSBP/PI4P pathway, (+)RNA viruses may recruit sterols for their genomic RNA replication via different mechanisms. For instance, replication proteins p33 and p92 of TBSV bind directly to sterols *in vitro* (Xu and Nagy, 2017). The cholesterol recognition/interaction amino acid consensus (CRAC) in p33 determines its sterol binding activity and a substitution in the CRAC domain abolishes TBSV replication in both yeast and plant cells (Xu and Nagy, 2017). TBSV also modulates the host VAP protein Scs2p to form MCSs, leading to enrichment of sterols at the viral replication sites in both yeast and plant cells (Barajas et al., 2014). Examination of cellular lipid levels suggests that enriched sterols at Tombusvirus replication sites were transported from existing cellular pools because sterol synthesis is not increased in cells (Xu and Nagy, 2017). In addition to TBSV, WNV also redistributes cholesterol and cholesterol-synthesizing proteins to sites of viral replication (Mackenzie et al., 2007). However, the mechanism of this redistribution requires further investigation. In addition, disruption of certain enzymes involved in sterol biosynthesis, including 3-hydroxy-methyglutaryl-CoA reductase (Mackenzie et al., 2007), *ERG25*- and *ERG4*-encoded proteins (Sharma et al., 2010), mevalonate diphospho decarboxylase (Rothwell et al., 2009), and 24-dehydrocholesterol reductase (Takano et al., 2011), inhibits replication of WNV, TBSV, DENV, and HCV, respectively, suggesting the involvement of sterols in the replication of a group of diverse (+)RNA viruses.

What are the possible roles of enriched sterols in (+)RNA viruses replication? The direct binding of sterols to viral replication proteins, such as TBSV p33 and p92 (Xu and Nagy, 2017), suggest that sterol-enriched cellular membranes may facilitate the recruitment of viral proteins and proviral host factors to VRCs or aid in the exclusion of other host components from the rearranged membranes. Sterols also intercalate into phospholipid bilayers (Demel and De Kruyff, 1976) and thus, might facilitate the assembly or function of VRCs.

## Fatty Acids in (+)RNA Virus Replication

Fatty acids (FAs) are basic building blocks for the majority of cellular lipids, including PLs, SLs, and neutral lipids (Figure 1). They are involved in multiple processes of cellular growth and development through their roles in transcriptional regulation, signaling transduction, and post-translational modification of cellular proteins (Moellering and Benning, 2011; Troncoso-Ponce et al., 2013; Quilichini et al., 2015). Cellular FAs may be generated from *de novo* synthesis, lipid hydrolysis, protein de-lipidation, or external sources. In yeast cells, FAs are originally synthesized in the cytosol and mitochondria and then transferred to the ER for elongation and saturation (Klug and Daum, 2014). However, in plants, *de novo* synthesis of FA occurs in plastids and the majority of long chain FAs are transported to ER membranes for further modification, including elongation and acyl editing (Li et al., 2016).

FAs have been reported to play crucial roles in the replication of multiple (+)RNA viruses. Applying pharmacological inhibitors to disrupt FA synthase (FASN) inhibits the replication of DENV (Heaton et al., 2010), yellow fever virus (Heaton et al., 2010), and WNV (Heaton et al., 2010; Martin-Acebes et al., 2011). It was further found that nonstructural protein 3 (NS3) of DENV interacts with and recruits FASN to viral replication sites to produce FAs (Heaton et al., 2010). In DENV-infected host cells, NS3 enhanced FASN enzymatic activity but not the amount of FASN protein (Heaton et al., 2010). For HCV, FA synthesis was promoted by an increase in both FASN protein level and activity (Nasheri et al., 2013) or an increased amount of acetyl-CoA synthetase (Kapadia and Chisari, 2005). Conversely, HCV RNA replication was inhibited 3-fold by the acetyl-CoA carboxylase inhibitor TOFA, which decreases cellular FA synthesis (Kapadia and Chisari, 2005).

FAs consist of different species depending on the chain length and degree of saturation (or unsaturation) of the hydrocarbon tails. The saturation degree of FAs affects genomic replication of (+)RNA viruses, likely through modulating membrane flexibility. For example, certain saturated and monounsaturated FAs promote HCV replication but some polyunsaturated FAs inhibit HCV replication, suggesting that balanced FA composition is needed for efficient HCV replication (Kapadia and Chisari, 2005). BMV requires high levels of UFAs to support its genome replication in yeast cells (Lee et al., 2001; Lee and Ahlquist, 2003). A single point mutation in *OLE1* allows host cells to grow normally but inhibits BMV replication by more than 20-fold (Lee et al., 2001). Further investigation demonstrated that VRCs are still formed but UFAs are locally depleted at the VRC-associated membranes, indicating that the lipid composition of VRC membranes differs from the rest of ER membranes (Lee and Ahlquist, 2003). Disrupting the expression or activity of mammalian stearoyl-CoA desaturase-1 (SCD1), which converts SFAs to monoUFAs, also inhibits HCV replication, suggesting the importance of UFAs in the replication of a group of (+)RNA viruses (Lyn et al., 2014; Nguyen et al., 2014). Similar to BMV, addition of SCD1 products (monoUFAs) can bypass the requirement of SCD1, indicating that SCD1 activity rather than physical presence is crucial for HCV replication (Nguyen et al., 2014). In contrast to the role of UFAs in contributing to the function but not formation of BMV VRCs, a high concentration of SCD1 inhibitor blocks the formation of HCV VRCs (Lyn et al., 2014).

The level of FA saturation, or the UFA/SFA ratio, is critical for membrane-associated functions because of their strong effects on membrane fluidity and other related properties (Emmerson et al., 1999). Membrane fluidity plays crucial roles in activating protein functions or modulating protein-protein and protein-membrane interactions (Kinnunen et al., 1994). Thus, reduced UFA levels might perturb the formation and stability of interactions among viral and host factors, disrupting the formation and maintenance of proper membranous structure used in viral replication. Nevertheless, the clear mechanism of this process needs further investigation.

The degree of saturation is not the only factor involved in how FAs affect viral replication, as illustrated in BMV VRC formation and genome replication (Zhang et al., 2012). Acyl-CoA binding protein (ACBP) is a long-chain fatty acyl-CoA binding protein that stimulates the activity of acetyl-CoA carboxylase and FASN, both of which are involved in FA synthesis (Rasmussen et al., 1993; Faergeman and Knudsen, 1997). ACBP is encoded by a single gene *ACB1* in yeast. ACBP is required for BMV replication because deletion of *ACB1* results in a more than 10-fold reduction in BMV genome replication and the formation of abnormal VRCs, that are smaller in size but greater in number compared to those in wt cells (Zhang et al., 2012). The UFA/SFA ratio increased 33%, from a ratio of 4.5 in wt cells to 6 in *acb1*Δ cells. However, the increased UFA is only partially responsible for BMV replication defects, suggesting more research is required to identify responsible factors, such as other lipid species or proteins whose conformation is affected by lipid compositional changes (Zhang et al., 2012). Interestingly, a group of BMV 1a mutants (termed Class II mutants) a phenocopied deletion of *ACB1*, including aberrant VRCs that are smaller but more numerous than those induced by wt BMV 1a, among several others (Liu et al., 2009). Each of the four BMV 1a Class II mutants has a single amino acid substitution in an amphipathic α-helix of BMV 1a, termed helix A. The amphipathic α-helix inserts into membranes, with the hydrophobic half inserted into acyl tails and the hydrophilic half interacting with head groups of PLs (Drin and Antonny, 2010). Consistent with the property as an amphipathic α-helix, Helix A is required for the perinuclear ER membrane association of BMV 1a (Liu et al., 2009). The similar phenotypes caused by lipid compositional alterations or by substitutions within membrane-anchoring helix of BMV 1a, confirm the notion that protein-lipid/membrane interactions govern the rearrangement of membranes and the formation of VRCs. Further delineation of the interactions should provide more specific contributions from host or viral side to the rearrangement of cellular membrane during viral replication.

## Lipid Droplet and (+)RNA Virus Replication

Lipid droplets (LDs) serve as essential depots for storage of lipids (SLs, FAs, and PLs) and energy. The LD core is mainly composed of StEs and TAG, which is surrounded by a phospholipid monolayer predominately constituted of PC and PE (Figure 3) (Tauchi-Sato et al., 2002). The surface of LDs is embedded with some specific proteins that are involved in lipid metabolism. LDs can either be formed *de novo* from ER or from fission of existing LDs (Jacquier et al., 2011, 2013). They move through the cytoplasm dynamically and interact with other cellular organelles, including ER (Martin and Parton, 2006), to facilitate the transport of lipids and proteins among organelles. LDs are utilized by some (+)RNA viruses as the sites for virion assembly (Roingeard and Melo, 2017), beyond the synthesis of viral RNA, and will not be discussed in this review.

Due to its lipid core, LDs are exploited by (+)RNA viruses to acquire lipids for membrane or energy production to support their replication. DENV stimulates lipophagy, a specialized autophagy process targeting LDs for degradation and to mobilize the stored lipids as free FAs (Singh et al., 2009). During DENV replication, free FAs generated by lipophagy are produced. However, the FAs from LDs during DENV replication are not used for membrane synthesis but primarily processed in mitochondria via β-oxidation to produce ATP, which supports efficient DENV genome replication (Heaton and Randall, 2010). Addition of exogenous FAs in growth media bypassed the requirement of autophagy for DENV replication in a β-oxidation-dependent manner, implicating crucial roles of FAs and energy production via FA breakdown during DENV replication (Heaton and Randall, 2010). It was later shown that DENV replication protein NS4A was responsible for the induction of autophagy (McLean et al., 2011), and host AMP kinase-mTOR signaling pathway was required for DENV-stimulated lipophagy (Jordan and Randall, 2017). However, the mechanism by which DENV stimulates lipophagy was not clear until the elucidation of a critical role for host AUP1 (ancient ubiquitous protein 1) in DENV-promoted lipophage and viral infection (Zhang J. et al., 2018). In mock-infected cells, AUP1 localizes to LDs, is mono- and oligo-ubiqutinated, and has low acyltransferase activity. During DENV infection, AUP1 is relocalized to autophgagosomes, de-ubiquitinated, and has significantly enhanced acyltransferase activity. All of the above AUP1's features require its interaction with DENV NS4A and are necessary for the DENV-promoted lipophagy. While AUP1 is not required for the induction of autophagy in general, it is specifically necessary for the DENV-promoted lipophagy. However, it is not known what AUP1 does upon moving to autophagosomes and how it acts on LDs. Surprisingly, knockout of *AUP1* blocked DENV virion production but not replication, which differs from what had been previously reported (Heaton and Randall, 2010; McLean et al., 2011). The difference may be because in general only lipophagy, but not autophagy, was blocked when AUP1 was knocked out (Zhang J. et al., 2018). Nevertheless, in AUP1 knockout cells, DENV structural protein (E-protein) was preferentially retained in the ER and degraded. AUP1 is also involved in ER-associated degradation (Klemm et al., 2011). It remains to be seen whether AUP1's main role in DENV infection is primarily involved in E-protein modification and stability or in relation to lipophagy, or both.

To promote PC synthesis for making VRCs, poliovirus not only translocates CCTα from nuclei to the sites of polioviral replication but also targets LDs to acquire FAs (Viktorova et al., 2018). Compared to mock-infected cells, the number of LDs dropped and fluorescently labeled long-chain FAs were firstly found to be incorporated into LDs but later at poliovirus replication sites (Viktorova et al., 2018). In comparison to DENV, which takes advantage of lipophagy for energy, poliovirus recruits, and enriches lipases to LDs to release FAs for stimulating PC synthesis. Two lipases are recruited and enriched at LDs to release free FAs: hormone sensitive lipase (HSL), which is translocated from its primary location of perinuclear areas, and adipocyte triglyceride lipase (ATGL) (Viktorova et al., 2018). However, the mechanisms whereby HSL and ATGL are recruited remain to be determined.

## Conclusions, Cautions, and Future Directions

With emerging novel technologies (such as fluorescent probes for lipid detection and mass spectrometry-based lipidomics), lipids have been demonstrated to play key roles in almost every stage of the viral life cycle. In this review, we focused on crucial roles that lipids play in genomic replication of (+)RNA viruses. As summarized in Table 1, different viruses may require the same lipid class for their efficient viral genomic RNA replication. For instance, PC and sterols are required for the replication of multiple viruses from different families, implying that pathways involved in PC and sterol accumulation at the replication membranes are potential targets to develop broad-spectrum antiviral drugs. In fact, some existing FDA-approved drugs target FA synthesis (e.g., FASN) and thus, inhibit replication of some FA-requiring viruses, including DENV, WNV, and HCV that are discussed in this review (Table 1). Therefore, research into the relationship between lipid metabolism and (+)RNA virus replication will uncover potential targets to develop broad-spectrum antiviral products.

Many research groups have identified some specific lipid species that are required for the replication of corresponding viruses (Figure 2 and Table 1). For example, PE and PC are required for the replication of TBSV and BMV, respectively, and the replication proteins of these two viruses modulate specific lipid synthesis pathways to facilitate viral genomic RNA replication. However, caution should be taken when generalizing results between virus classes and host organisms as the specific lipids may only be one part of a complex system with many factors and alternative solutions. There are different lipid biosynthesis pathways that are present, absent, or predominate in different cell-types and organisms. The composition of membranes varies among yeast, plant, insect and mammalian cell types, and one should consider that there may be multiple combinations of specific lipids that allow for efficient viral replication. FHV provides an interesting example, as its replication complexes can be re-targeted to different intracellular membranes (e.g., from outer mitochondrial membrane to ER) yet still retain RNA replication capabilities (Miller et al., 2003). This suggests some promiscuity in viral requirements for membrane composition. Another example is that some viruses can form VRCs of different morphology by varying the expression levels of replication proteins (Schwartz et al., 2004). If specific lipids help to “shape” VRCs, then how does the lipid composition of these alternative VRCs compare to wt composition? How these lipids are involved in viral replication processes, such as transcriptional activation, regulation of gene expression, modification of proteins, assembling of functional VRCs and/or some other key steps remain to be elucidated.

Currently, the majority of reports hypothesize the potential roles of lipids in the assembly of VRCs. However, how lipids are organized in VRCs and their roles in the assembly and maintenance of VRCs still needs to be further investigated. Fluorescent imaging has been used to observe lipids in relation to other cellular components and viral replication sites. However, conventional fluorescent microscopy techniques, such as confocal microscopy, pose limitations due to low spatial resolution, and the inability to image subcellular components in full detail. Super-resolution microscopy has the ability to precisely localize single fluorescent molecules with a precision of ~10–20 nm, depending on the sample and the instrument. This could provide a novel way of imaging lipids and subcellular organelles, leading to a better understanding of the trafficking and recruitment of certain lipids in (+)RNA virus replication. In addition, super-resolution microscopy has the potential to be used in live-cells, providing insight into the formation of VRCs upon viral infection. The precision of super-resolution microscopy allows for a more detailed picture of the localization and movement of lipids, providing further insight into their role in the replication of (+)RNA viruses.

In conclusion, despite the complexity of lipid metabolism and virus-host interactions, knowledge of how viruses modulate host lipid synthesis pathways and remodel cellular membranes to facilitate replication, will undoubtedly unmask many new directions in cell biology, and accelerate the process of developing antiviral strategies.

## Author Contributions

ZZ, GH, and NF collected references and drafted the manuscript. ZZ made all figures. ZZ, NF, BK, GB, GR, and XW revised the manuscript.

### Conflict of Interest Statement

The authors declare that the research was conducted in the absence of any commercial or financial relationships that could be construed as a potential conflict of interest.
